# Computational methods for identifying enhancer‐promoter interactions

**DOI:** 10.15302/J-QB-022-0322

**Published:** 2023-06-01

**Authors:** Haiyan Gong, Zhengyuan Chen, Yuxin Tang, Minghong Li, Sichen Zhang, Xiaotong Zhang, Yang Chen

**Affiliations:** ^1^ School of Computer and Communication Engineering Beijing Advanced Innovation Center for Materials Genome Engineering University of Science and Technology Beijing Beijing 100083 China; ^2^ State Key Laboratory of Medical Molecular Biology Department of Biochemistry and Molecular Biology Institute of Basic Medical Sciences School of Basic Medicine Chinese Academy of Medical Sciences Peking Union Medical College Beijing 100005 China; ^3^ Shunde Innovation School University of Science and Technology Beijing Foshan 528399 China

**Keywords:** enhancer, promoter, enhancer‐promoter interaction, machine learning, deep learning

## Abstract

**Background:**

As parts of the cis‐regulatory mechanism of the human genome, interactions between distal enhancers and proximal promoters play a crucial role. Enhancers, promoters, and enhancer‐promoter interactions (EPIs) can be detected using many sequencing technologies and computation models. However, a systematic review that summarizes these EPI identification methods and that can help researchers apply and optimize them is still needed.

**Results:**

In this review, we first emphasize the role of EPIs in regulating gene expression and describe a generic framework for predicting enhancer‐promoter interaction. Next, we review prediction methods for enhancers, promoters, loops, and enhancer‐promoter interactions using different data features that have emerged since 2010, and we summarize the websites available for obtaining enhancers, promoters, and enhancer‐promoter interaction datasets. Finally, we review the application of the methods for identifying EPIs in diseases such as cancer.

**Conclusions:**

The advance of computer technology has allowed traditional machine learning, and deep learning methods to be used to predict enhancer, promoter, and EPIs from genetic, genomic, and epigenomic features. In the past decade, models based on deep learning, especially transfer learning, have been proposed for directly predicting enhancer‐promoter interactions from DNA sequences, and these models can reduce the parameter training time required of bioinformatics researchers. We believe this review can provide detailed research frameworks for researchers who are beginning to study enhancers, promoters, and their interactions.

## Abstract 2

**Author summary:** In this review article, we discuss prediction methods for enhancers, promoters, and enhancer‐promoter interactions that may help researchers choose methods for identifying cis‐regulatory elements.

## INTRODUCTION

It is known that cis‐acting regulatory elements (CREs) are DNA sequences that have transcriptional regulatory functions in the human genome. An enhancer (20‐ to 400‐bp) [1] is a class of non‐coding DNA sequences bound by transcription factors [2], and these sequences can interact with short regions of DNA (100–1000 bp), known as promoters, located near the gene transcription start sites (TSS) of a gene [3]. Enhancers and promoters are essential cis‐regulatory elements for promoting gene transcription activities over a long distance. The interactions between distal enhancers (even with tens of kilobases) and proximal promoters regulate target genes and inhibit the cis‐regulatory mechanism of the human genome [4, 5, 6, 7, 8, 9].

Studying the mechanism of enhancer and promoter interactions (EPIs) may help us to understand the regulatory relationships among genes and reveal the genes associated with diseases. Davison *et al.* showed that EPIs can lead to type I diabetes and multiple sclerosis, and that new genes related to these diseases can be predicted using EPIs [10]. Smemo *et al.* [11] found the first intron region of the *FTO* gene in mice and humans, and the homologous gene *IRx3* was found to exist in a distal EPI. In the human brain, heart, and lungs high levels of *IRx3* gene are expressed; this is very important for controlling weight. Therefore, the study of EPIs, especially cell line‐specific EPIs, may provide insight into the mechanisms of gene expression regulation, cell differentiation, and disease. In addition, research on EPIs has provided new methods and ideas for diagnosing and treating disease as well as for developing drugs.

Many sequencing technologies have been developed to generate data and identify enhancer, promoter, and chromosome interactions. For example, epigenomic features such as the histones and transcription factor binding sites (TFBS) data generated by chromatin immunoprecipitation (ChIP‐seq) [12,13] and cleavage under targets and release using nuclease (CUT&RUN) [14] technologies have been widely used to identify enhancers and promoters. High‐throughput chromosome conformation capture (Hi‐C) [15] data (such as BL‐Hi‐C [16]) is frequently used to call loops (chromosome interactions that connect two distal regulatory elements). Promoter Capture Hi‐C [17], Chromatin Interaction Analysis with Paired‐End‐Tag sequencing (ChIA‐PET) [18], and HiChIP [19] can also identify genomic features such as enhancer‐promoter interactions. Genetic features such as DNA sequences, pseudo dinucleotide composition (PseDNC), and Pseudo k‐tuple nucleotide composition (PseKNC) [20] are also widely used to predict enhancers and promoters. Although the amount of high‐throughput sequencing data is increasing rapidly, there are few enhancer‐promoter interaction datasets that have been validated by experiments. The prediction of enhancer‐promoter interactions using machine learning, deep learning, or other methods is therefore one of the most promising research topics in bioinformatics.

Numerous review articles have been published in recent decades concerning: enhancer interactions, including their role [21] at the genome‐wide level; transcription enhancers in animal development, evolution [22], and disease [23]; functional contributions to transcription [24,25]; the functional significance of enhancer chromatin modification [26]; models that describe dynamic three‐dimensional chromosome topology related to development enhancers; methods for identifying enhancer target genes [27] and enhancers [28, 29, 30]; the mechanisms of EPIs in higher eukaryotes [31]; bioinformatics analysis methods related to EPIs prediction [32, 33, 34, 35]; analysis from sequence data [36,37]; and how EPIs control gene expression [38]. However, with the advancement of computational methods in the past decade, research has increasingly proposed methods for detecting enhancer‐promoter interaction tools based on traditional machine learning or deep learning, but there has yet to be a global overview of solutions specifically for EPI identification.

In light of this issue, this paper proposes computational models for identifying enhancer‐promoter interactions based on high‐throughput experimental data published from 2010 to 2022. First, we discuss the relationship between EPIs and gene transcription, and we provide a general framework for enhancer‐promoter identification. Next, we discuss in detail recognition methods that have been developed in the last decade for enhancers and promoters, chromatin loops, and enhancer‐promoter interactions; we summarize available enhancer and promoter resources, and suggest realistic guidelines for their use. Finally, we review the application of methods for identifying EPIs in diseases such as cancer.

## REGULATION OF GENE EXPRESSION VIA EPIs

**Figure 1:**
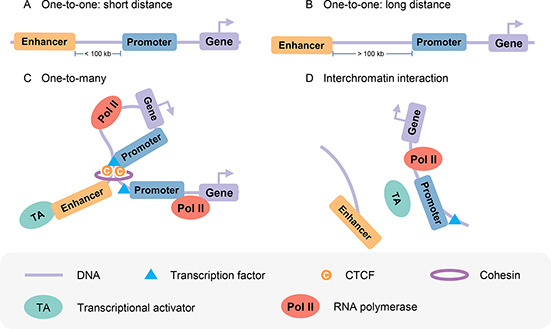
**Mechanisms of transcriptional activation over EPIs.** (A, B) Enhancers activate transcription from target promoters over a short distance or long distance. (C) One enhancer interacts with many promoters. (D) Interchromosomal enhancer and promoter interaction.

Previous studies [39, 40, 41] have shown that the intrachromosomal and interchromosomal communications between enhancer and promoter regulate gene transcription. Transcription from target promoters can be activated by enhancers in interchromatin or intrachromatin over a short distance or a long distance (more than 100 kb) [1] ( Fig.1, B), and one enhancer may interact with multiple promoters ( Fig.1). He *et al*. [42] observed that the number of targets for each promoter is 2.92 on average. Some transcription factors may also mediate the interchromosomal interaction between enhancer and promoter ( Fig.1). For example, Patel *et al*. [43] found a T‐cell‐specific cis‐regulatory element in chromosome 16 (TIL16) that can interact with the *TAL1* promoter through interchromosomal interaction, and *c‐Maf* and *p300* may cooperate to mediate the interchromosomal loop for abnormal activation of *TAL1* in T‐ALL cells. Therefore, the prediction of enhancers, promoters, and their interactions is vital to our understanding of gene transcription mechanisms.

**Figure 2:**
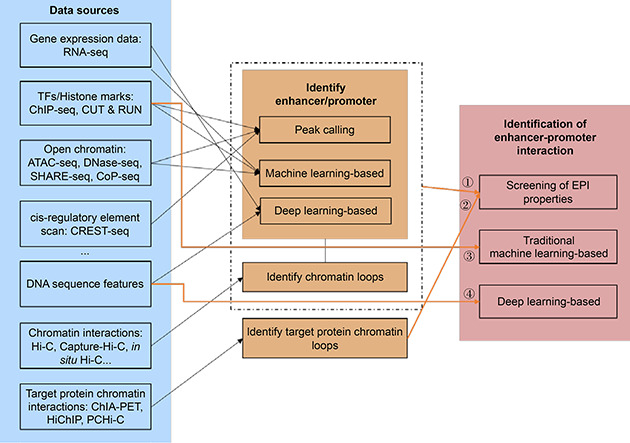
An overview of the EPI prediction methods using different data sources.

EPIs can be identified by formulating the problem as follows: “Given two DNA sequences (A and B) described by different data types, first, determine if either A or B can function as an enhancer or a promoter, then determine if A and B are a chromatin loop”. A general process for identifying EPIs is shown in Fig.2, which shows that the identification of EPIs can be divided into four categories:

(i) Given two DNA sequences with transcription factors (TFs), histone marks (provided by ChIP‐seq), and chromatin interactions (provided by Hi‐C) information, we first need to determine whether the given two DNA sequences are enhancers or promoters by calling peaks, or methods based on traditional machine learning, deep‐learning. Then, we need to call loops from Hi‐C data to determine whether the two DNA sequences form a chromatin loop.

(ii) Given two DNA sequences with protein chromatin interaction information (provided by ChIA‐PET, HiChIP, or PCHi‐C), we can call chromatin loops to determine whether the two DNA sequences have EPIs.

(iii) Given two DNA sequences with TFs, histone marks features, and other epigenomic features, two DNA sequences can be identified as EPIs or not by machine‐learning‐based methods.

(iv) Given two DNA sequences without other information, the two DNA sequences can be identified as EPIs or not by deep‐learning‐based methods.

Thus we see that the data analysis process can be categorized into the prediction of enhancers, promoters, and EPIs. In the following sections, we describe the prediction of enhancers and promoters, and the identification of EPIs, separately.

## PREDICTION OF ENHANCER AND PROMOTER

**Figure 3:**
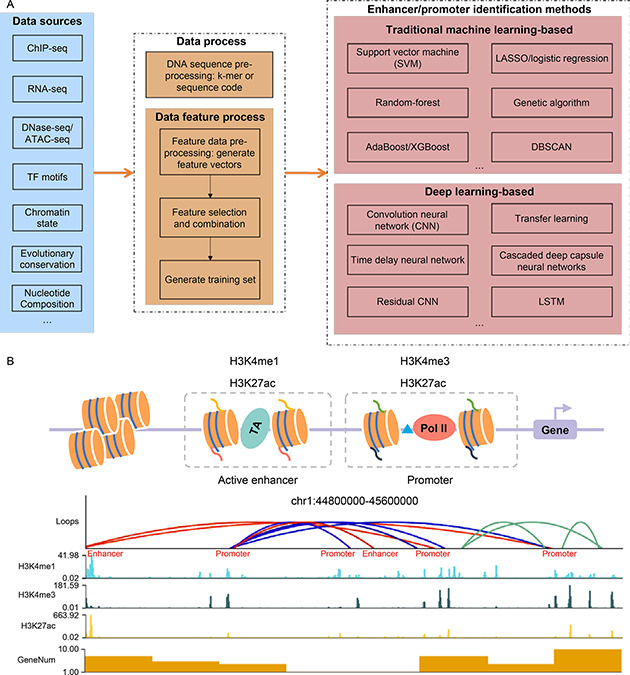
**An overview of the enhancer and promoter identification process.** (A) EPI identification methods include data processing and identification methods. (B) An example of identifying EPIs based on Hi‐C and ChIP‐Seq data. The red lines represent EPIs, the blue lines represent promoter‐promoter interactions, and the green lines represent other chromosome interactions.

As Tab.1, Tab.2, and Fig.3 show, we can choose methods based on traditional machine learning or deep learning to check if a given DNA sequence is an enhancer or a promoter. To do this, we first need to process the DNA sequence, generate a training set with labels (promoter, enhancer, or none), and then identify enhancers or promoters by traditional machine learning or deep learning.

**Table 1:** Prediction methods of enhancer

Category	Refs.	Time	Source data	Method	Software name	Citation number
Traditional machine learning‐ based	[44]	2011	DNA sequence	SVM (support vector machine) classifier	k‐mer‐svm	162
	[45]	2012	TF motifs	LASSO regression	CLARE	9
	[46]	2012	ChIP‐seq histone methylation and acetylation maps	Genetic algorithm‐optimized support vector machine	ChromaGenSVM	77
	[47]	2013	Histone modification ChIP‐seq	Random‐forest‐ based	RFECS	144
	[48]	2014	Gapped k‐mer features	SVM	gkm‐svm	239
	[49]	2014	Histone modifications (ChIP‐Seq), TFBSs, chromatin accessibility (DNase‐Seq), transcription (RNA‐Seq), evolutionary conservation, sequence signatures	Linear SVM and multiple kernel learning	EnhancerFinder	162
	[50]	2015	ChIP‐seq	AdaBoost‐based	DELTA	31
	[51]	2015	Histone ChIP‐seq and DNA sequence	SVM	DEEP	73
	[52]	2016	DNA sequence	Machine learning	iEnhancer‐PsedeKNC	15
	[53]	2016	DNA sequence, pseudo k‐tuple nucleotide composition	SVM	iEnhancer‐2L	334
	[54]	2016	Chromatin state, DNA sequence	A two‐step wrapper‐ based feature selection method	EnhancerPred	52
	[55]	2016	WGBS DNA methylation profiles	Weighted support vector machine learning framework	LMethyR‐SVM	9
Traditional machine learning‐ based	[56]	2017	Short dinucleotide repeat motifs (DRMs), DNA sequence, enhancer‐associated histone modification data	Machine learning	−	22
	[57]	2017	Chromatin state, DNA sequence	A two‐step wrapper‐based feature selection method	EnhancerPred2.0	29
	[58]	2017	Histone ChIP‐seq and methylation, DNA sequence	Random forest	REPTILE	43
	[59]	2018	DNA sequence	SVM	iEnhancer‐EL	106
	[60]	2018	FANTOM5 atlas of TrEns	Feature matrix generation, feature ranking using Gini‐index, logistic regression	TELS	2
	[61]	2018	DNA sequence	k‐mer and machine learning based method	enhancer_prediction	13
	[62]	2020	STARR‐seq	Supervised machine‐learning	MatchedFilter	21
	[20]	2021	DNA sequence	Feature extraction technique and SVM	piEnPred	6
	[63]	2021	Chromatin state and DNA sequence	Enhanced feature representation using random forest	iEnhancer‐RF	8
	[64]	2021	Nucleotide Composition	Two‐Layer Predictor, Kullback‐Leibler divergence, LASSO, SVM	iEnhancer‐KL	1
	[65]	2021	DNA sequence	7‐mer and random forest	Computational CRISPR Strategy (CCS)	38
	[66]	2021	DNA sequence	Random forest, extremely randomized tree, multilayer perceptron, SVM and extreme gradient boosting	Enhancer‐IF	12
Deep learning‐ based	[67]	2010	Histone modification ChIP‐seq	Time delay neural network (TDNN)	CSI‐ANN	160
	[68]	2016	ChIP‐Seq, DNase‐Seq, RNA‐Seq, DNA meth‐ ylation, and other features	Deep learning‐based	PEDLA	91
	[69]	2017	DNA sequence	CNN (convolution neural network)	DeepEnhancer	76
	[70]	2017	DNA sequence	Deep‐learning‐based	BiRen	91
	[71]	2018	ATAC‐Seq	Neural network‐based model	PEAS	22
	[72]	2019	DNA sequence	Word embeddings and SVM	iEnhancer‐5Step	96
	[73]	2020	DNA sequence	Word Embedding and CNN	iEnhancer‐CNN	26
	[74]	2021	DNA sequence and DNase‐seq	Deep‐learning‐based	DeepCAPE	8
	[75]	2021	STARR‐seq	Deep‐learning‐based	DECODE	2
	[76]	2021	DNA sequence	Augmented data and Residual CNN	ES‐ARCNN	4
	[77]	2021	Pseudo ‐ K‐tuple nucleotide composition and DNA sequence	DNN	iEnhancer‐DHF	8
	[78]	2021	DNA sequence	Word embedding, generative adversarial net, CNN	iEnhancer‐GAN	8
	[79]	2022	DNA sequence	Neural network	RicENN	1
	[80]	2022	DNA sequence	Enhanced feature extraction strategy, deep learning	−	0
	[81]	2022	DNA sequence	One‐hot encoding, convolutional neural network	iEnhancer‐Deep	2
	[82]	2022	DNA sequence	DBSCAN, random forest, word2vec and attention‐based Bi‐LSTM	−	0

**Table 2:** Prediction methods of promoter

Category	Refs.	Time	Source data	Method	Software name	Citation number
Deep learning‐ based	[83]	2012	DNA sequence	DNA sequence features	−	63
	[84]	2016	DNA sequence	Deep feature selection, DFS		200
	[85]	2017	DNA sequence	CNN	CNNProm	169
	[86]	2018	DNA sequence	SVM	BacSVM+	9
	[87]	2018	DNA sequence	DNA sequence features	iPromoter‐2L	256
	[88]	2019	DNA sequence	CNN and LSTM	DeePromoter	80
	[89]	2019	DNA sequence	Deep learning and combination of continuous FastText N‐Grams	deepPromoter	46
	[90]	2019	DNA sequence	Deep learning	PromID	68
	[91]	2019	DNA sequence	Minimum redundancy maximum relevance (mRMR) algorithm and increment feature selection strategy, SVM	iProEP	99
	[92]	2019	DNA sequence	Combinee smoothing cutting window algorithm, k‐mer, SVM	iPromoter‐2L2.0	57
	[93]	2019	Bacterial σ70 promoter sequences	Feature subspace based ensemble classifier	iPromoter‐FSEn	30
	[94]	2019	Bacterial σ70 promoter sequences	Multiple windowing and minimal features	iPro70‐FMWin	20
	[95]	2019	The physicochemical properties of nucleotides and their nucleotide density into pseudo K‐tuple nucleotide composition	A two‐layer predictor	iPSW(2L)‐PseKNC	55
	[96]	2019	DNA sequence	F‐score feature selection method	MULTiPly	87
	[97]	2020	DNA sequence of *Escherichia coli* K‐12	Statistical physics model	PhysMPrePro	1
	[98]	2020	DNA sequence of *Escherichia coli* K‐12	CNN	iPromoter‐BnCNN	23
	[99]	2020	DNA sequence of *Escherichia coli* K‐12	CNN, pseudo‐di‐nucleotide composition	PseDNC‐DL	32
	[100]	2020	DNA sequence of *Escherichia coli* K‐12	One‐hot encoding and CNN	pcPromoter‐CNN	17
	[101]	2021	The k‐mer nucleotide composition, binary encoding and dinucleotide property matrix‐based distance	Extremely randomized trees	iPromoter‐ET	5
	[102]	2021	Rice‐specific DNA sequence	CNN	Cr‐Prom	9
	[103]	2021	DNA sequence of *Escherichia coli* K‐12	A two‐layer predictor	iPro2L‐PSTKNC	5
	[104]	2021	DNA sequence	CNN	iPTT(2 L)‐CNN	2
	[105]	2021	DNA sequence	Cascaded deep capsule neural networks	Depicter	23
	[106]	2022	DNA sequence	k‐mers and deep learning network	PPred‐PCKSM	1
	[107]	2022	DNA sequence	k‐mer word vector, multiple descriptors and feature selection using XGBoost	dPromoter‐XGBoost	1
	[108]	2022	DNA sequence	k‐mers and LSTM network	−	1
	[109]	2022	DNA sequence	Moran‐based spatial auto‐cross correlation method and deep convolution generative adversarial network	iPro‐GAN	2
	[110]	2022	Promoter data sets from both plants and humans	Synthetic sampling, transfer learning and label smoothing regularization	HMPI	0
	[111]	2022	Promoter sequences from six nannochloropsis strains	Densely connected convolutional neural networks	DenseNet‐PredictPromoter	0
Peak calling	[112]	2015	Capture Hi‐C	−	−	861
	[113]	2016	Promoter capture Hi‐C	−	−	769

### Vector representations of DNA sequences

To generate the DNA sequence vectors that can be recognized by traditional machine learning or deep learning, we first need to code the DNA sequence ( *e.g.,* ATCGGC…) in one of the following ways. (i) One‐hot encoding, although has two problems: (1) the curse of dimensionality and (2) the distance between any pair of one‐hot vectors is equal. (ii) To overcome the two problems of one‐hot encoding, we use a word embedding algorithm, such as Word2vec [114] or Glove [115], to encode the DNA sequence. For example, dna2vec [116] first transforms a sequence into k‐mers (a DNA sequence of length k) and then transforms the k‐mers into vectors using Word2vec.

#### The training sets for enhancers and promoters

There are two ways to obtain enhancer and promoter training sets: (i) Download data sets from a public data repository. For example, we can download the human and mouse enhancer data sets from the SEDB [117] database and can download the eukaryotic promoter from the EPD [118] database. More databases for enhancers and promoters are listed in Tab.3. (ii) Available research has shown that H3K4me1 and H3K27ac enrichment occurs in both enhancers and promoters and that H3K4me1 together with H3K27ac, and a lack of H3K4me3 at the same genomic site can distinguish enhancers from promoters [49]. Additionally, enhancers are enriched with TFBS, Med1. Therefore, we can identify enhancers and promoters by calling peaks from TFBS, H3K27ac, H3K4me1, H3K4me3, or Med1 ChIP‐seq data. As Fig.3 shows, we downloaded H3K27ac, H3K4me3, and H3K4me1 ChIP‐seq data in the Hela‐S3 cell line from the ENCODE platform under accession number ENCSR000AOC, ENCSR000AOF, and ENCSR000APW, respectively. The genome sites with H3K27ac, H3K4me3, and H3K4me1 ChIP‐seq signals were identified as promoters. The genome sites with H3K27ac, H3K4me1 ChIP‐seq signals, but without H3K4me3 signals were identified as enhancers.

#### Methods for identifying enhancer/promoter based on traditional machine learning

In machine learning‐based methods, the enhancer/promoter identification problem can be reformulated into a binary classification problem (yes or no). Since 2010, support vector machine (SVM) [20,44,46,48,49,51,53,55,59,64,66,86,91,92,140,141], regression [45,60], random forest [47,58,63,65,66,101], boost‐based [50,66], and other traditional machine learning methods [52,56,61,62,83,84,87,93, 94, 95, 96,103] have all been applied to predict enhancers and promoters. The SVM‐based method combined with feature selection has been the most used, even within the last three years. For example, the kmer‐SVM [44] first finds the motif related to enhancers by k‐mer analysis, then inputs the motif into the SVM model to get the classification results. piEnPred [20] takes advantage of feature extraction techniques such as k‐mer, composition of k‐spaced nucleic acid pairs (CKSNAP), Dinucleotide‐based cross covariance (DCC), PseDNC, and PseKNC to extract features and SVM to classify enhancers and promoters.

**Table 3:** Available public data repository for enhancer and promoter

Database type	Data repository name
Enhancer	Sedb [117]
	PReMod [119]
	Human Transcribed Enhancer Atlas [120]
	VISTA [121]
	dbSUPER [122]
	ENdb [123] (human enhancer)
	SEA [124]
	RAEdb [125]
	SELER (human cancers) [126]
	EnDisease [127]
	dbInDel [128]
	CancerEnD (cancer associated enhancers) [129]
	CPE‐DB [130]
	Animal‐eRNAdb [131]
Promoter	EPD [118]
	PlantProm (plant promoter) [132]
	TransGene Promoters, TGP [133]
	Osteo‐Promoter Database (OPD) skeletal cells [134]
	Osiris [135]
	TiProD [136]
	PromoterCAD (mammalian promoter/enhancer) [137]
	EPDNew [138]
	PPD [139]

Generally, there are three steps to traditional machine learning‐based methods. (i) Use of feature extraction techniques to extract features [20,54,57,60,63,84,91,93,96], such as gene expression, histone modification marks, DNA sequence features, and TFs motifs. (ii) Classification of enhancers and promoters by classification algorithms, such as SVM, random forest, or regression. (iii) Tuning of the model parameters and optimization of the target functions using optimization algorithms, such as genetic algorithms [46].

After surveying the accession and citation numbers of these traditional machine‐learning methods ( Tab.1), we recommend that users who do not want to run code using the web server iEnhancer‐2L [53] should identify enhancers and their strengths using pseudo k‐tuple nucleotide composition. For users who want to run code by themselves, we recommend that they choose gkm‐svm [48], REPTILE [58], and CCS [65]. These tools provide detailed information and example data for users to get up to speed and run them quickly.

#### Methods for identifying enhancer/promoter based on deep‐learning

Methods based on deep‐learning primarily focus on training a neural network with DNA sequences or DNA sequences with epigenomic characteristics (such as histone modifications, chromatin accessibility, DNA methylation, or CpG islands) as inputs. Though some scholars have trained their networks with epigenome features [67,68,71,74,75,82], most have done so with only DNA sequences as inputs [69,70,72,73,77, 78, 79, 80, 81,85,88, 89, 90,98, 99, 100,102,104, 105, 106, 107, 108, 109, 110, 111,142]. Predicting enhancers and promoters directly from DNA sequences is believed to be more applicable than identifying them from multiple epigenomic features because the epigenomic characteristics data carries with it substantial sequencing costs, and a high rate of false positives. However, prediction methods that use epigenomic characteristics in their inputs are more accurate than those that only use DNA sequences.

Methods based on deep‐learning can be roughly divided into the following two steps. (i) Encoding a DNA sequence as in Section “Vector representations of DNA sequence”. (ii) Constructing a neural network to predict the presence of enhancers or promoters, such as CNN [69,73,76,78,81,85,88,98, 99, 100,102,104,111], transfer learning [110], or LSTM [82,88,108]. To establish the right characteristics and increase the accuracy of identifying an enhancer or promoter, the above methods either improve the input layer of DNA feature vector representation (for example, dna2vec) or neural network architectures or change the activation functions. Tab.1 and Tab.2 list the available deep‐learning‐based methods for detecting enhancers and promoters. CSI‐ANN [67] was the first deep learning‐based method for the identification of enhancers, though Yang *et al.* [78] have since proposed iEnhancer‐GAN to identify enhancers using word embedding, generative adversarial net, and CNN to capture DNA sequence features.

Although computational methods such as traditional machine learning and deep learning have achieved solid results, some problems still exist. One problem is that such methods typically use gene expression data such as chromatin characteristics and histone modification information as features to train models. When gene expression data are missing, these models cannot predict enhancers. Another problem is that enhancers are species‐specific. That is, enhancers are expressed differently by different species, so the current methods have low performance in predicting enhancers across species.

For these deep‐learning‐based methods, we give some suggestions for tool selection. For users who want to predict using ChIP‐seq, RNA‐seq data, and other features as inputs, we recommend methods based on the input data requirements. For users who wish to identify enhancers and promoters with only DNA sequences as inputs, the number of citations metric ( Tab.1 and Tab.2) shows that BiRen [70] and PromID [90] are used frequently for predicting enhancers and promoters, respectively. Online tools including ES‐ARCNN [76], iEnhancer‐Deep [81] and iPromoter‐2L [87] are easy to use and return the prediction results from these methods quickly.

## PREDICTION OF ENHANCER‐PROMOTER INTERACTION

The task of recognizing EPIs is based on the prediction of enhancers and promoters individually in order to determine if there is an interaction between them, and this is a challenging task. First, multiple promoters can be activated by one enhancer, and multiple enhancers can coordinate to regulate one promoter. Secondly, EPI has tissue‐specificity [42]. These features result in poor generalization for current EPI recognition methods. The existing EPI recognition methods are divided into three main types: (i) screen EPIs based on high‐throughput sequencing experiments, (ii) methods based on traditional machine‐learning, and (iii) methods based on deep‐learning.

### Generation of EPIs training sets

In surveying the benchmarking EPI data sets used in 12 EPI identification methods ( Tab.4), we found 10 methods used the EPI data sets in GM12878, HUVEC, Hela‐S3, IMR90, K562, and NHEK cell line proposed by TargetFinder [143]. TargetFinder integrates TFs, histone markers, Dnase‐seq, gene expression, and DNA methylation data to predict EPIs. However, before training any model, the EPI data sets need to be augmented, such as with the synthetic minority oversampling technique [156], because of the low ratio of positive to negative data sets (1/35). There are two ways to generate an acceptable EPI dataset.

(i) We can label the active enhancer and promoter regions using ChIP‐seq data or annotation files and then annotate chromosome interactions from Hi‐C data. For example, EPIP [154] obtained the enhancer data sets and identified the promoter data sets from transcription start site (TSS) annotation files by considering the genomic regions between the 1000 bases upstream and 100 bases downstream of the TSS regions. We can also obtain enhancer and promoter data sets from databases listed in Tab.3. To train an EPI identification model, we can divide the training dataset into positive and negative EPI data sets by overlapping the training data set with the regions of the loops called from Hi‐C data [15]. For example, EPIP [154] states that if an enhancer and a promoter overlap with a pair of regions from loops within 30 reads, this pair of enhancer and promoter is considered a positive EPI. We can then use the loop callers listed in Tab.5 to call loops from Hi‐C data, such as HiCCUPS [157], HiGlass [159], cLoops [160], FitHiC2 [161], Mustache [162], and HiC‐ACT [164]. As Fig.3 displays, to show how to identify EPIs, we downloaded the Hi‐C data from 4dnucleome platform under accession number 4DNESCMX7L58, called loops using Mustache [162], and then annotated these loops as enhancer‐promoter interactions or promoter‐promoter interactions based on ChIP‐seq signals.

**Table 4:** The benchmarking enhancer‐promoter interaction dataset used in EPIs identification methods

EPI dataset	EPIs methods that used the dataset
EPI Dataset provided by Whalen *et al*. [143]	PEP [144], EP2vec [145], SPEID [146], random forest based method [147], Zhuang *et al.* [148], EPIVAN [149], Singh *et al.* [150], EPI‐DLMH [151], EPIsHilbert [152], EPI‐Mind [153]
Dataset provided by Talukder *et al.* [154]	EPIP [154]
Dataset provided by Jing *et al.* [155]	SEPT [155]

(ii) We can also obtain EPI data sets by screening loops from target proteins HiChIP, PLAC‐seq, or ChIA‐PET data. For example, first, H3K27ac HiChIP data can be used to identify enhancer regions by calling loops. Then, we can screen loops that interact with promoters as EPIs. Many available loop callers have been developed for HiChIP, PLAC‐seq, and ChIA‐PET data. As Tab.5 shows, tools such as HiC‐Pro [158], hichipper [166], MAPS [169], FitHiChIP [167], and HiChIP‐Peaks [170] have been developed for HiChIP and PLAC‐seq data, and tools like ChIA‐PET Tool [171], MICC [173], ChIA‐PET2 [175], ChIAPoP [176], ChIA‐PIPE [177], and MACPET [178] have been developed for ChIA‐PET data. Among these tools, HiC‐Pro [158] is a pipeline tool for analyzing Hi‐C data that includes data pre‐processing and calling loops, and FitHiChIP [167] is a fast and memory‐efficient loop caller for identifying significant loops. In addition, ChIA‐PET2 [175] identifies loops in raw ChIA‐PET sequencing reads of different types.

**Table 5:** Methods of calling loops from 3C‐based data

Publication	Time	Sequencing technology	Method	Software name	Citation number
[157]	2014	Hi‐C [15]	Identify “enriched pixels” where the interaction frequency is higher than expected	HiCCUPS	753
[158]	2015	Hi‐C, HiChIP	Toolkit	HiC‐Pro	1125
[159]	2018	Hi‐C		HiGlass	402
[160]	2020	Hi‐C, ChIA‐PET	DBSCAN‐based	cLoops	35
[161]	2020	Hi‐C	Identify loops from high‐resolution Hi‐C	FitHiC2	72
[162]	2020	Hi‐C, Micro‐C [163]	Scale‐space representation	Mustache	42
[164]	2021	Hi‐C	Aggregated Cauchy test	HiC‐ACT	10
[165]	2021	Hi‐C	Identify loops from high‐resolution Hi‐C	HiCORE	1
[166]	2018	HiChIP [19]	DNA loop calling	hichipper	86
[167]	2019	HiChIP/PLAC‐seq [168]	Jointly models the non‐uniform coverage and genomic distance scaling of contact counts	FitHiChIP	76
[169]	2019	HiChIP/PLAC‐seq	Zero‐truncated Poisson regression framework	MAPS	65
[170]	2020	HiChIP	Differential peak analysis	HiChIP‐Peaks	6
[171]	2010	ChIA‐PET	Automatic processing of ChIA‐PET data	ChIA‐PET Tool	308
[172]	2014	ChIA‐PET	A statistical model	chiasig	44
[173]	2015	ChIA‐PET	R package to detect chromatin interactions from ChIA‐PET	MICC	30
[174]	2015	ChIA‐PET	Hierarchical Dirichlet process	3CPET	21
[175]	2017	ChIA‐PET	Analysis pipeline	ChIA‐PET2	71
[176]	2019	ChIA‐PET	Analysis pipeline	ChIAPoP	5
[177]	2020	ChIA‐PET	Analysis pipeline	ChIA‐PIPE	8
[178]	2020	ChIA‐PET	Consider different noise levels in different genomic regions	MACPET	0

### Methods for identifying EPIs based on traditional machine‐learning

The development of high‐throughput sequencing technology has produced a huge amount of genomic information, relating to factors such as histone modification and chromatin accessibility. These factors data make it possible to recognize EPIs based on traditional machine learning methods. The basic idea is to use different high‐throughput genomic signals as input features of a traditional machine learning model to predict these interactions through statistical calculations. The TF and RNA polymerase ChIP‐seq have been reported to be the factors data that can detect EPIs by analyzing epigenomic signals in enhancers and promoters, including TargetFinder [143], EPIP [154], and the XGBoost‐based approach [179]. In recent years, boosting ensemble learning methods ( *e.g.,* Adaboost [180], gradient boosting decision tree (GBDT) [181], and XGboost [182]) have been used to predict EPIs by constructing multiple weak classifiers. For example, Yu *et al.* [179] first generated EPI data sets based on chromatin contact data, annotated histones and binding protein data, and a GTF file, and then extracted epigenomic and sequence features. They then trained the XGBoost‐based model by five‐fold cross‐validation in order to predict EPIs. They [179] showed that XGBoost performed better than other machine learning methods, such as TargetFinder [143], random forest [147,183], GBDT [145], or Adaboost [154].

Methods based on traditional machine learning have the advantage of high accuracy for predicting EPIs. However, they have not been widely used for two reasons. The first is the lack of epigenetic characteristics in many cell lines, and the second is that traditional machine‐learning‐based methods require researchers to possess professional knowledge of epigenetics and manually connect the interaction characteristics.

### Methods for identifying EPIs based on deep‐learning

With the development of deep learning, methods for identifying EPIs based on deep‐learninghave been proposed for building different neural network architectures in order to learn from DNA sequences without epigenomic characteristics. As is the case for the deep learning‐based methods for predicting enhancers and promoters, the process of predicting EPIs includes three steps: (i) embedding the promoter and enhancer DNA sequences based on one‐hot encoding or dna2vec, (ii) extracting the promoter and enhancer sequence features based on CNN, LSTM (long short‐term memory), or transformer learning, and (iii) predicting EPIs based on the trained network.

Zhuang *et al.* [148] used one‐hot to encode the DNA sequence of enhancers and promoters, but the data storage needed for one‐hot encoding consumes a great deal of computer memory and results in the loss of the association information among DNA sequences. EPIVAN [149] and EPI‐Mind [153] use dna2vec to embed k‐mer into a 100‐dimensional vector and contained more information than was the case for one‐hot encoding. Singh *et al.* [146] proposed SPEID to predict long‐range EPIs that combine CNN with LSTM. SPEID [146] first inputs the enhancer and promoter vectors encoded by one‐hot into CNN, fuses the high‐dimensional features extracted from the enhancer and promoter, inputs the fused features into LSTM, and finally outputs the prediction results through the full connection layer. SEPT [155], EPIsHilbert [152], TransEPI [184], and EPI‐Mind [153] used transfer learning to get more cross‐cell type data features automatically. With the development of deep learning technology, applying transfer learning to the identification of EPIs can reduce the parameter training necessary for each different cell line.

Lastly, we counted the number of citations for available EPI tools, and found that TargetFinder [143] and IM‐PET [42] were the most used EPI tools based on traditional machine‐learning methods and that EPIVAN [149] and SPEID [146] were the most used EPI tools based on deep‐learning methods. Though the web server EPIXplore [185] has not been cited by any article, we suggest that users who do not want to run code access EPIXplore, because EPIXplore integrates IM‐PET [42], EpiTensor [186], TargetFinder [143], JEME [187], and 3DPredictor [188], and provides downstream analysis as well as a visualization module. To explore the role that enhancer‐promoter interaction structures play in determining normal and pathogenic cell states, we need to use tools that can identify differential EPIs in a process similar to differential expression analysis. Although there is no way to identify differential EPIs directly, we can combine the identification tools for differential loops and EPIs. For example, Lareau *et al.* proposed diffloop [189] to identify differential loops from ChIA‐PET data and identified 1974 differential EPIs from 2 MCF7 and 2 K562 samples. diffHiC [190], FIND [191], HICcompare [192], multiHiCcompare [193], and Serpentine [194] all identify differential loops from Hi‐C data.

## APPLICATIONS OF METHODS FOR IDENTIFYING EPIs IN DISEASES

**Figure 4:**
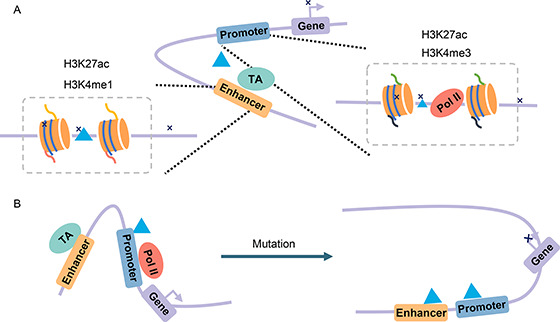
**Dynamic EPI affects gene transcription.** (A) Mutations at enhancers or promoters can lead to disease or to repressed gene expression. (B) The differential EPIs before and after cell mutation.

Genome‐wide association studies (GWAS) have revealed that noncoding regulatory sequences, especially the enhancer regions with strong cell specificity, are associated with disease variations [195,196]. Thus, any of the mutations that appear in enhancer‐promoter interactions may cause diseases. Carullo *et al*. [197] discussed in their review study that two types of mutations may disrupt transcriptional regulation ( Fig.4). First, the mutations of transcription factors or chromatin modifiers are found at enhancers. Marsman *et al.* [198] discussed the fact that the gene expression is regulated by transcription factors during cell development, and gene differentiation is regulated by changing loop conformations. For example, as Fig.4 shows, the kit gene is expressed by transcription factors ( *e.g*., GATA‐2) in immature erythrocytes, where the enhancers and kit promoter are linked via these transcription factors. When cells mature, other TFs ( *e.g*., GATA‐1) that bind to the downstream element (DE) take the place of the GATA‐2 TF. TFs including GATA‐1 mediate looping between the kit promoter and DE, leading to the disappearance of the loop between enhancer and promoter and the downregulation of kit. Li *et al.* [199] also showed that the GATA‐2 expression and DNA‐binding are important for the cell differential process. Second, the mutations of sequence located in enhancers may lead to the loss or gain of functions. Wang *et al.* [200] proposed the model APRIL to construct long‐range regulatory networks and predict novel disease‐associated genes with predicted enhancer‐gene interactions as inputs (for example, from JEME [187] or IM‐PET [42]). In a study by Rodin *et al.* [201], whole‐genome sequencing was performed on 59 donors with autism spectrum disorder (ASD) and 15 control donors and functional enhancers provided by IM‐PET [42] to demonstrate that ASD shows an excess of somatic mutations in neural enhancer sequences. Li *et al.* [18] suggested there is a possibility that mosaic enhancer mutations are associated with ASD risk. In addition, Fachal *et al.* [202] applied computational enhancer–promoter correlations (using IM‐PET [42] and FANTOM5 [60]) and a Bayesian approach (PAINTOR) that they proposed to finely‐map 150 breast cancer risk regions and identify 191 likely target genes.

## CONCLUSION AND FUTURE PROSPECTIVE

Computational methods for identification of enhancers, promoters, and EPIs are valuable for accelerating gene regulation studies, and this paper has reviewed the most important ones to come along over the past decade. We have proposed a basic framework for identifying EPIs and divided the identification methods of EPIs into the following two categories: (i) screening EPIs from ChIP‐seq, Hi‐C, HiChIP, ChIA‐PET, or other High‐throughput sequencing technology and (ii) identifying EPIs from DNA sequences, ChIP‐seq, Hi‐C, or other epigenome data by methods based on traditional machine learning or deep learning. This review also covered enhancer and promoter databases ( Tab.3), as well as methods of identifying enhancers ( Tab.1), promoters ( Tab.2), chromatin loops ( Tab.5), and enhancer‐promoter interactions ( Tab.6). These tables provide practical guidance for readers in selecting methods by model type or input data type in order to identify EPIs. We believe this review can serve as a foundational resource that allows researchers to apply traditional machine learning and deep learning methods to the prediction of enhancers, promoters, and EPIs in future research. We now summarize some important topics for this future work.

First, the initial step of EPI identification based on traditional machine‐learning or deep‐learning is to pre‐process the DNA sequences using one‐hot, k‐mer, or dna2vec algorithms. However, these methods do not maintain the spatial proximity of the sequence. Designing a new sequence coding method that can maintain the spatial proximity and sequence features is the next task that we urge the EPI research community to undertake.

**Table 6:** Prediction methods of enhancer‐promoter interaction (EPI)

Category	Refs.	Time	Source data	Method	Software name	Citation number
Traditional machine learning‐based + call loops from Hi‐C data	[42]	2014	DNA, histone marks, TFBSs, RNA‐seq, ChIA‐PET	Random forest	IM‐PET	242
	[143]	2016	ChIP‐seq, Hi‐C	Machine learning‐based	TargetFinder	349
	[154]	2019	Hi‐C, enhancer, and promoter DNA sequences, ChIP‐seq	Data screen, balanced and unbalanced models	EPIP	22
	[188]	2020	ChIP‐seq, RNA‐seq, Hi‐C	Machine‐learning‐based	3DPredictor	32
Traditional machine learning‐based	[203]	2017	ChIP‐Seq	Bayesian classifier	EP_Bayes	8
	[187]	2017	DHS, distance, eRNA, histone marks, ChIA‐PET/Hi‐C/eQTL	Linear regression	JEME	166
	[183]	2017	5C, FAIRE‐seq, ChIP‐seq, Cap‐analysis gene expression (CAGE), DNA methylation, nucleosome occupancy, eRNAs, chromatin state	Random forest classifier	−	11
	[144]	2017	DNA sequence	Gradient boosting	PEP	67
	[204]	2018	DNA structure properties and transcription factor binding motifs	Machine‐learning‐based	−	3
	[145]	2018	DNA sequences of arbitrary lengths	Natural language processing and unsupervised deep learning (extract sequence embedding feature), GBDT	EP2vec	56
	[147]	2019	ChIP‐seq	Random forest	−	2
	[179]	2020	DNA sequence, ChIP‐seq, annotation file	XGBoost‐based	XGBoost	11
	[205]	2022	CT‐FOCS	Linear mixed effect models	ct‐focs	2
Deep‐learning‐based	[148]	2019	DNA sequence	CNN and a recurrent neural network	EPIsCNN	38
	[146]	2019	DNA sequence	CNN, LSTM	SPEID	94
	[149]	2020	DNA sequence	Dna2vec, deep‐learning‐based	EPIVAN	100
	[155]	2020	Hi‐C, ChromHMM of Roadmap Epigenomics	CNN, transfer learning	SEPT	14
	[151]	2021	DNA sequence	CNN, bidirectional gated recurrent unit network and matching heuristic mechanism	EPI‐DLMH	18
	[152]	2021	Hi‐C, DNA sequence,	Hilbert curve encoding, transfer learning	EPIsHilbert	2
	[184]	2022	Hi‐C, ChIA‐PET	Transformer‐based model	TransEPI	1
	[153]	2022	DNA sequence	Dna2vec, transfer learning	EPI‐Mind	0
	[185]	2022	−	A web server for prediction EPI	EPIXplorer	0

Secondly, although traditional machine‐learning and deep‐learning methods have furthered bioinformatics studies for enhancers, promoters, and EPIs for the past ten years, the precision of traditional machine learning is limited because of the high complexity of the source data, its features, and its limited possible model combinations. With recent increases in computing power, however, deep‐learning‐based methods for identifying EPIs directly from DNA sequences without other epigenome data features have begun to be developed. Furthermore, the rise of transfer learning has reduced the parameter training time needed by bioinformatics researchers. One model can even be fine‐tuned by using transfer learning and then transferred to other models for training, which can significantly reduce the amount of needed calculations. For example, transfer learning can be used to predict EPIs [152,153,155,184] across cell lines. An appropriate model trained in one cell line can then be used to predict EPIs directly in another cell line, and this is something that we believe should become a research priority in the future.

Thirdly, with the development of single cell sequence technology, EPI studies at the single‐cell level can help us solve the problem of cell heterogeneity, and analyze the mechanism and relationship between individual cells and the body. To accomplish this, available EPI identification methods need to be optimized to accommodate the sparsity of single‐cell sequencing data, such as scATAC‐seq, scHi‐C.

Fourthly, the application of EPI identification methods to exploring tumor‐specific EPIs, the effect of mutations on EPIs, and the relationship between EPI formation and gene expression remains the central problem in EPI research. With the development of CRISPR technologies (CRISPR/Cas9, CRISPRa, CRISPRi) and CRISPR screening (Perturb‐seq, CRISPRi‐FlowFISH etc.), we are now able to identify EPIs or assess the role of EPIs in specific tumors and gene regulatory systems.

## ABBREVIATIONS

**Table:** 

CREs	cis‐acting regulatory elements
EPI(s)	Enhancer‐promoter interaction(s)
TSS	Transcription start sites
ChIP‐seq	Chromatin immunoprecipitation
CUT& RUN	Cleavage under targets and release using nuclease
Hi‐C	High‐throughput chromosome conformation capture
ChIA‐PET	Chromatin interaction analysis with paired‐end‐tag sequencing
TFs	Transcription factors
TFBS	Transcription factor binding sites
CKSNAP	Composition of k‐spaced nucleic acid pair
DCC	Dinucleotide‐based cross covariance
PseDNC	Pseudo dinucleotide composition
PseKNC	Pseudo k‐tuple nucleotide composition
SVM	Support vector machine
CNN	Convolution neural network
GBDT	Gradient boosting decision tree
LSTM	Long short‐term memory
DE	Downstream element

## COMPLIANCE WITH ETHICS GUIDELINES

Haiyan Gong, Zhengyuan Chen, Yuxin Tang, Minghong Li, Sichen Zhang, Xiaotong Zhang, and Yang Chen declare that they have no conflict of interest.

This article is a review article and does not contain any studies with human or animal subjects performed by any of the authors.
